# Supplementary material for: A Swiss-Roll-Type Methanol Mini-Steam Reformer for Hydrogen Generation with High Efficiency and Long-Term Durability
Source: Micromachines (Basel). 2023 Sep 27;14(10):1845. doi: 10.3390/mi14101845 (PMC10608973; doi:10.3390/mi14101845)
Supplement: Supplementary file 1 [file micromachines-14-01845-s001.zip › micromachines-2544038-supplementary.pdf]

A Swiss-Roll type Methanol Mini-Steam Reformer for Hydrogen Generation with High Efficiency and Long-Term Durability

Fan-Gang Tseng[1] * ,Wei-Cheng Chiu[1] and Po-Jung Huang[1]
[1] Department of Engineering and System Science, National Tsing Hua University, TAIWAN
[2]Department of Energy Engineering, National United University, TAIWAN
* corresponding: fangang@ess.nthu.edu.tw

1. The results of CFD analysis

Table S1. Velocity, pressure and temperature differences of the tube type and Swiss-roll reformers from simulations

type	Tube type	Swiss roll
Velocity(average)	0.142[m/s]	0.133[m/s]
pressure(average)	70.3176[Pa]	76.3361[Pa]
temperature(average)	486.292[k]	489.584[k]
Velocity(difference)	0.181[m/s]	0.262[m/s]
pressure(pressure drop)	140.542[Pa]	152.717[Pa]
temperature(difference)	1.8[k]	0.5[k]

Table S2. Boundary conditions and Governing equations for flow simulation in ANSYS

<table>
<tr><th colspan="3">CFD Boundary conditions</th></tr>
<tr><th></th><th>Mass flow rate</th><th>Pressure</th></tr>
<tr><td>inlet</td><td>300 sccm</td><td rowspan="2">Atmosphere pressure</td></tr>
<tr><td>outlet</td><td></td></tr>
</table>

<table>
<tr><th colspan="2">CFD Boundary conditions</th></tr>
<tr><td>wall</td><td>Non slip wall on wall channel</td></tr>
<tr><td>Fluid</td><td>Methanol vapor</td></tr>
<tr><td>S/C ratio</td><td>1.2</td></tr>
<tr><td>Diffusion temperature</td><td>523°K</td></tr>
</table>

Governing equations	
**Porous media simulation**	$s_i = -\left(\Sigma_{j=1}^{3}\frac{u}{\alpha}v_i + \Sigma_{j=1}^{3}C_2\frac{1}{2}\rho\lvert v\rvert v_i\right)$ where s_i is the source term, v is for velocity, α is the permeability, μ is for viscosity

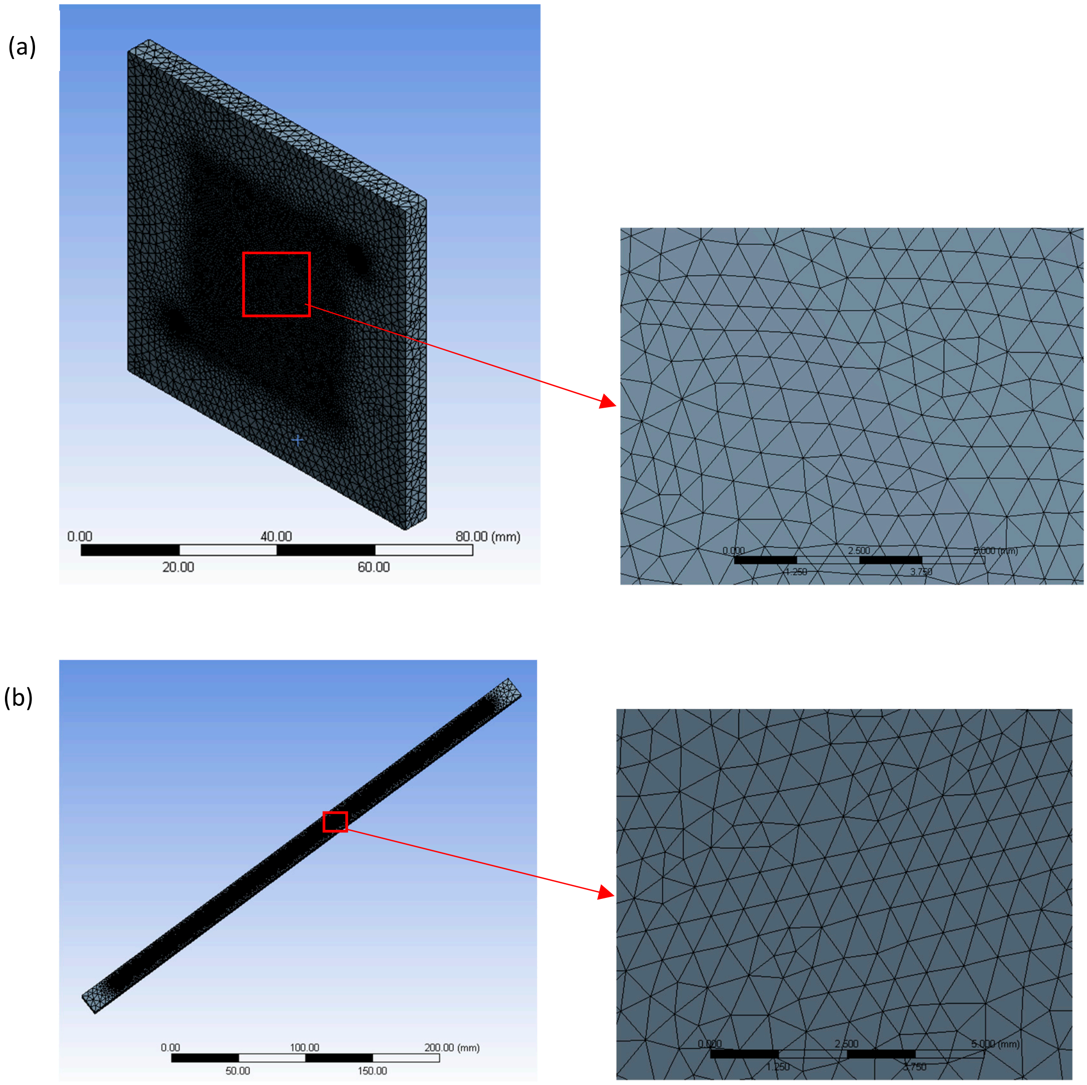

Figure S1. The mesh distributions for reformer and temperature measurement (a) Whole swiss roll, (b) Whole tube type flow

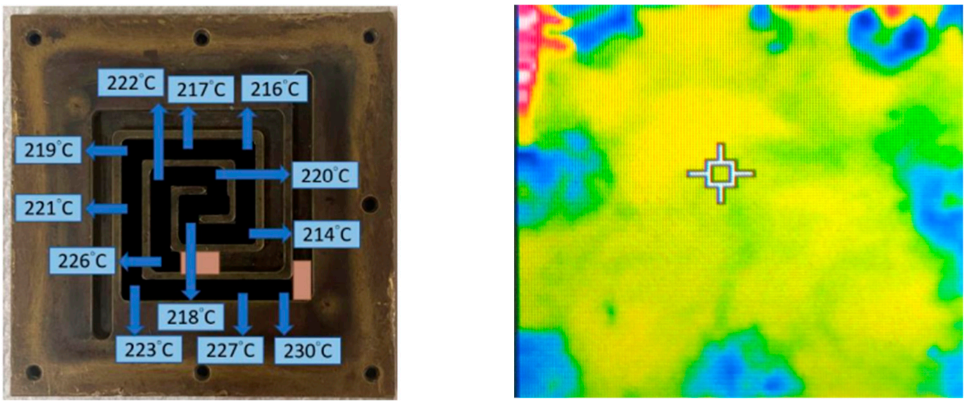

Figure S2. Thermal couple measure point and value, infrared thermography image

The presence of eight relatively cooler spots near the periphery in the thermal image is attributed to the passage of long screws through the reformer, which was not included in the simulated model. Apart from this, the temperature distribution in the central part of the reformer exhibits a comparable uniformity in both the measured and simulated results.

1. Catalyst weight and GHSV

Table S3. The relationship of catalyst weight and GHSV

Catalyst weight (g)	1	1.25	1.5	1.75
GHSV (ml/(g·h))	29167.98	23334.38	19445.32	16667.42
Catalyst weight (g)	2	2.25	2.5	
GHSV (ml/g·h))	14583.99	12963.54	11667.19	
